# Retraction Note: SETDB1 promotes glioblastoma growth via CSF-1-dependent macrophage recruitment by activating the AKT/mTOR signaling pathway

**DOI:** 10.1186/s13046-022-02495-y

**Published:** 2022-09-21

**Authors:** Shuai Han, Wei Zhen, Tongqi Guo, Jianjun Zou, Fuyong Li

**Affiliations:** 1 Department of Neurosurgery, the First Hospital of China Medical University, Shenyang, Liaoning Province China; 2 Department of Neurosurgery, The People’s Hospital of China Medical University (The People’s Hospital of Liaoning Province), No.33, Wenyi Road, Shenhe District, Shenyang, 110016 Liaoning Province PR China

**Retraction Note: J Exp Clin Cancer Res 39, 218 (2020)**

**https://doi.org/10.1186/s13046-020-01730-8**

The Editor in Chief has retracted this article. After publication it was noted that there were irregularities in figures 1A, 2H, 3A, B, D, E, 6A and 7C, when the authors were contacted regarding these issues they were unable to provide the original data. The authors were also unable to provide any evidence of ethics approval for this article.

Fuyong Li agrees to this retraction. Shuai Han, Wei Zhen, Tongqi Guo and Jianjun Zou have not responded to any correspondence from the editor/publisher about this retraction.

